# Gerhard on the Epidermic Administration of Medicine

**Published:** 1830

**Authors:** 


### II. Analytical.

1. *Gerhard on the epidermic administration of Medicine.*—The peculiar speculations of the physicians of the physiological school have lately made this mode of administering medicine very popular, and as Dr. G. in the capacity of resident physician to the Philadelphia Almshouse, has had ample opportunities of testing the value of the practice we will present our readers with an abstract of a paper published by him in the N. Amer. Med. and Surg. Jour.

The violent constitutional effects occasionally following the application of poisonous agents to ulcerated surfaces have so long been familiar to the profession, that it is rather a matter of surprise that among the infinite variety of novelties, both practical and speculative, which are constantly claiming the attention of the profession, this mode of administering medicine without subjecting the patient to the inconvenience of tasting it, should not at an earlier period, have been more extensively employed.

Numerous circumstances may render the administration of medicine, per vias naturales inconvenient, sometimes impracticable, of these not the least in the estimation of the Brousaians, is the danger of producing irritation or inflammation of the mucous surface of the intestines. The local irritation which always accompanies or precedes intermittent fever is frequently such as to prevent the administration of the preparations of the bark when the system would be greatly benefitted by their use. In dropsical effusions preceded by inflammatory action, such is the proneness of the mucous surface to take on inflammation, that drastic purgatives, and even ordinary diuretics are precluded. Under these circumstances, medicines may be applied to the denuded cutis with as much confidence as by the usual mode of administration. Narcotics too are frequently applied with success, when opium could not be borne in any of the usual modes of administration, or when the stomach has become so habituated to its impression that it no longer acts as an anodyne.

Those medicines are best adapted for this mode of administration, which are unirritating, easily soluble, and whose active properties are concentrated in a small space. The more active vegetables, or their extracts, and some of the tinctures may be used with great propriety. Powerful chemical irritants on the one hand, and substances of little activity on the other, cannot be applied with any prospect of advantage.

Immediately after the application of an active agent to the cutis, considerable pain is the consequence. This local irritation is independent of the specific effects of the medicine, and tends to counteract it; and hence the most irritating remedies are seldom successful when applied in this way. Thus tartar emetic and other mineral substances afford very unsatisfactory results; croton oil and elaterium frequently fail while aloes purges actively.

As to the mode of application, dry substances may be sprinkled upon the part; but if moist and not easily pulverized, they may be reduced to a proper consistence, and spread upon the surface, or upon a piece of linen and applied to it. Fluids may be used as a lotion, or applied through the medium of a compress. Oily substances, and sometimes powders which are too irritating to be applied directly, may be mixed with cerate.

Cantharides are usually preferred for removing the cuticle. They are continued until sufficient irritation is produced, and their action completed by the application of warm poultices. The size will depend upon the nature of the agent ; a surface of four inches square is usually found sufficiently large. At the first dressing a small portion of the cuticle may be raised, and the medicine applied under it ;—at a subsequent dressing, the cutis becomes less irritable, and supports the action of the remedy with less pain.

The energy with which medicines act upon the system through the cutaneous tissue, depends much upon the vascularity and sensibility of the part to which they are applied, and upon its vicinity to the centre of circulation. The epigastrium is the most suitable place ; though the anterior part of the neck and thorax, and the interior portions of the thighs and arms answer very well. The back and the extreme parts of the limbs do not possess sufficient powers of absorption, or sympathies with the vital organs sufficiently intimate, to be relied upon.

Corpulency, pregnancy, or great debility are unfavorable to the successful application of medicine in this way, and the temperament may be so irritable as to preclude the practice entirely. With respect to the dose, double or treble the quantity is usually necessary, and a much larger proportion may usually be born without inconvenience. Some medicines however, under favorable circumstances, operate as powerfully as when received into the stomach.

*Tonics.* The sulp. of quina when applied to the inflamed cutis, Dr. G. remarks produces considerable pain, lasting from a few minutes to a much longer period, according to the susceptibility of the patient. In a few hours the customary tonic effects of the medicine are visible. The dry red tongue which is frequently produced by the internal administration of tonics, rarely follows the endermic administration of quina.

Twenty cases of intermittent fever were treated by the endermic application of this remedy, and with perfect success, except in three or four cases, in which accidental circumstances required its discontinuance. The blister was always applied during the apyrexia, and the application of the remedy, as under other circumstances, avoided during the paroxysm. In twelve cases the disease did not return after the first application, there was rarely more than one paroxysm, and a third in but a single instance. The average quantity applied was about twenty grains. It was generally remarked that the cure was more speedy than when the medicine was administered internally. After the paroxysms were arrested, the internal administration of the quina was resorted to prevent their recurrence.

A number of cases are detailed of which our limits will permit us to give but one.

*Case* 3. Mary Lonton, aged 20. Temperate habits. Admitted November 11th. Nine days since she was attacked with fever, which soon assumed an intermittent type. Has a chill daily at eleven o'clock, A. M. November 11th. Full, tense pulse, headach, &c. Ordered venesection oz xvi. seidlitz powders. 12th. Some fever during the interval ; acet. ammon. Chill at eleven o'clock, venesection oz xiv. during the fever. Afternoon : no headach, cool skin, sulph. quina one grain every three hours during night, and every two hours next morning. 13th. Chill at the usual time ; paroxysms less violent, apyrexia tolerably perfect ; no headach, cool skin, pulse 86. Quina, one grain every hour. 14th. Chill again, but of rather shorter duration ; seidlitz powders. Complains of tightness at the epigastrium. Ordered cups ; and the quinia every two hours. 15th. Tightness at the epigastrium returned. Missed her chill for the first time. In the afternoon she was found in convulsions, to which she is subject, and which are always preceded by a sensation of tightness : pulse firm ; venesection oz xvi. sinapisms : enemeta of senna tea. 16th. Pulse 104 ; feels better, no uneasiness at the epigastrium, cool skin : sinapism ; dry cups to the back of the neck. Chill at the usual time, at eleven, P. M. pulse 86. Quinia every two hours during night. 17th. Chill returned with headach, pulse 94, dry skin ; stop quinia. At half past ten o'clock, A. M. Dr. Jackson saw the patient, and directed a blister to the epigastrium, and quinia to be used as an endermic application : six grains were applied at eight P. M., six grains during the night, and the same quantity next morning. 18th. Pulse active ; headach, but diminished in the evening : quinia continued ; no chill. 19th. Slept well, scarcely any pain in the head ; pulse 104, cool skin ; moist, but slightly furred tongue. No chill at the usual time, but returned at seven P. M., with some headach ; no tightness at the epigastrium ; quinia dressings continued in the former quantity. 20th. Restless night; less headach, but complains of pain from the application: bowels costive; quinia reduced to two grains three times a day, and mass. hydrarg: gr. v. rhei. pulv. gr. xv. *m.* to be given in pills: no chill. 21st. Very slight chill at seven P. M.; continue applications. 22d. Slept well; no headach; cool skin; moist tongue; pulse 80; bowels open; she has no chills; pulse natural; a little tightness at the epigastrium; no change in treatment. 24th. Tightness at the epigastrium; pulse 80; skin cool; blister nearly dry; quinia now suspended. At noon a convulsion came on: the usual remedies were employed, and the disease was perfectly removed in the evening. No medicines were administered after this period, as the patient was entirely convalescent, and recovered without another unpleasant symptom. This case is very interesting, as it illustrates the great practical advantage to be derived from the endermic treatment of intermittents. Although the quinia had been given with the greatest caution, all the symptoms were materially aggravated; but the same remedy applied externally, even in an irritable subject, immediately converted the quotidian into a mild tertian, and in a short time effected an entire cure. Another patient in the ward at the same time, was treated with the most perfect success in the manner just described, after a fruitless internal administration of the ordinary medicine.

The character of the fever is not sufficiently defined to permit us to comment upon the treatment. The most of our readers, we think, however, will be struck with the freedom with which the lancet was used, and the substitution of seidlitz powders and cups, for the purgatives generally used where the physiological doctrines have not come in vogue. The inference in favor of the endermic treatment drawn from this case, may justly be questioned; since if the unfavorable effect of the first administration of the quina arose from visceral congestion accompanied with inflammatory action, the copious venesection, and the interval of two days, after the first administration of the remedy, may have had a considerable influence in preparing the system for the administration of tonics. Without questioning the judgment of the Philadelphia physicians, we think that a Doctor of the old school, by convulsing the organs by the perturbating powers of calomel and scammony, would justly have calculated upon relieving this epigastric tightness, in a much shorter time, and by remedies, which to the majority of patients would appear much more simple.

The extract of cinchona as prepared by the Messrs. Wetherills, reduced to the semifluid state, effected in one case a very speedy cure.

*Narcotics.* The application of the different preparations of opium as a local application, has long been practised, and when administered with a view to their local effects, they have frequently been observed to bring the whole system under their influence. Powdered opium sprinkled upon a blistered surface, very soon manifests its specific effects. The dregs remaining after the preparation of the tincture, when united with cataplasms as a soothing ingredient for painful ulcers, frequently produce a constitutional impression so decided as to require their suspension. The tincture on account of the irritation it produces is inferior to the aqueous solution, and both are inferior to the black drop.

The sulphate and acetate of morphia exhibit the best form for the administration of this drug—since they possess great activity and may generally be applied without producing more than slight temporary irritation. The former is perhaps preferable, as the latter, under peculiar circumstances of great irritability, is liable on account of its greater insolubility to produce considerable pain. The morphia formed into a cerate, or perhaps more advantageously in the form of powder, in a quantity varying from 1 grain to 3 or 4 grs. according to the susceptibility of the patient may thus be applied. Dr. G. has collected a number of cases shewing that it may thus be advantageously used in intermittent fever, phthisis and other pulmonary affections, gonorrhea, neuralgia, rheumatism, and above all delirium tremens.

It is proper to notice that the endermic application of opium is sometimes followed by the same unpleasant after effects which result from its internal administration.

Satisfactory experiments have also been made with other narcotics. Six grains of the powdered digitalis sprinkled upon a blistered surface once a day, in one case reduced the pulse in a few days, from 120 to 75 strokes in a minute.

*Purgatives.* The operation of this class of medicines seems so intimately connected with the direct stimulation, (or more properly irritation) of the intestinal membrane, that the facility with which many of them operate when applied to the surface, is certainly calculated to excite the surprise of the profession. Aloes is the best adapted for this mode of application. It produces little irritation, and operates in nearly the same dose as when taken internally. Gamboge operates with considerable activity, but is objectionable on account of its frequently producing great irritation. Other purgatives were applied with unsatisfactory results. Even croton oil was ineffectual.

*Diuretics.* Dropsical effusions are so frequently confined with inflammatory symptoms, that the administration of this class of medicines requires great caution. The endermic mode of application therefore becomes an object of much importance. Dr. G. in a few instances, has applied squills both alone, and in combination with digitalis, with a very good effect.

*Emetics.* All the vegetable emetics, when applied to blistered surfaces, operate readily. As all the active property of ipecacuanha is concentrated in emetine, it may be used with great advantage. Three minims of the oil of tobacco having been applied to a blister, a violent vomiting came on in a few minutes after its application, which after continuing through the night was with difficulty arrested. The local irritation produced by emetics from the mineral kingdom, generally prevents their specific effects.

The facility with which mercury produces its specific effects through the skin is well known. Even the more acrid preparations are said to have the same effect. Iodine which is also a very irritating substance, affects the system as readily as mercury.

Dr. G. concludes his essay with the following inferences.

1. That medicines applied to various parts of the body externally, provided they be placed in direct contact with the vascular surface of the cutis, produce similar effects in doses but little larger than when they are made to act directly upon the gastric mucous membrane.

2. All medicinal substances have a peculiar affinity for certain organs or tissues, which is entirely independent of their immediate action upon such parts.

3. That violent irritants or escharotics rarely produce any general effect, although this sometimes occurs.

4. All other articles of sufficient activity may be used, provided the cutis be not too highly inflamed ; when the latter is the case, soothing applications are in the first place necessary, or no absorption will take place.

5. The endermic medication is peculiarly useful in cases of extreme gastric irritability, and when it is important to modify morbid actions without impairing the integrity of a vital organ.

*N. Amer. Med. and Phys. Jour.*

